# The use of CAM providers and psychiatric outpatient services in people with anxiety/depression: a cross-sectional survey

**DOI:** 10.1186/s12906-016-1446-9

**Published:** 2016-11-11

**Authors:** Anne Helen Hansen, Agnete E. Kristoffersen

**Affiliations:** 1 University Hospital of North Norway, PO box 35, , 9038 Tromsø, Norway; 2 Faculty of Health Sciences, Department of Community Medicine, UiT-The Arctic University of Norway, Tromsø, Norway; 3 The National Research Center in Complementary and Alternative Medicine (NAFKAM), Faculty of Health Sciences, Department of Community Medicine, UiT-The Arctic University of Norway, Tromsø, Norway

**Keywords:** Complementary and alternative medical providers, Psychiatric specialist services, Mental health care, Health care utilisation, Cross-sectional study, Norway

## Abstract

**Background:**

Depression has been identified as one of the most frequent predictors of CAM use. However, limited data exist about the use of CAM providers among people with anxiety/depression in Norway. The aim of this study was to investigate the use of CAM providers, and the use of CAM providers and psychiatric outpatient services in combination, among people with self-reported anxiety and/or depression.

**Methods:**

We used questionnaire data from 12,982 participants (30–87 years) in the cross-sectional sixth Tromsø Study (conducted in 2007-8). Eligible for analyses in our study were 1685 participants who reported suffering from anxiety and/or depression. By descriptive statistical methods, we estimated the use of CAM providers, psychiatric outpatient services, and the combination of these. By logistic regressions we studied the association between the use of these services and gender, age, income, education, and self-reported degree of anxiety/depression.

**Results:**

During the previous year, 17.8 % of people with anxiety/depression visited a CAM provider once or more, 11.8 % visited psychiatric outpatient services, and 2.5 % visited both. Men with anxiety/depression were less likely to visit CAM providers compared to women (odds ratio [OR] 0.40, confidence interval [CI] 0.30–0.55), whereas higher educated people were more likely to visit compared to the lowest educated (OR 1.47, CI 1.02–2.13). The use of CAM providers was not associated with the degree of anxiety/depression. For those who used both CAM providers and psychiatric specialist services during the previous year, severe anxiety/depression was strongly associated with use compared to moderate disease (OR 7.53, CI 2.75–20.65).

**Conclusions:**

People with severe anxiety/depression seem to use CAM providers and psychiatric services additionally, whereas those with moderate disease seem to use these services more as alternative pathways. CAM provider treatment might be a substitute for conventional care, particularly in patients with moderate disease.

## Background

Anxiety and depression are common mental disorders and global leading causes of all non-fatal burden of disease [1]. Nevertheless, around half of depressed persons worldwide do not receive treatment [2]. Most mental disorders emerge before the age of 30 [3, 4], and lack of treatment might contribute to disability for many crucial years of an individual’s life. In Norway, the 12 months prevalence of anxiety and depression is around 15 and 10 %, respectively [5].

Anxiety and depression are identified as strong predictors for use of complementary and alternative medicine (CAM), and self-directed CAM modalities are more widely used than therapies requiring consultations with a CAM practitioner [6–8]. Although use of CAM among patients with anxiety and depression seems common, limited data exist about the use of CAM providers and the combined use of CAM providers and psychiatric outpatient services among people with anxiety/depression in Norway.

In this study, CAM use is limited to the use of CAM providers and does not include the use of CAM techniques or products without CAM provider visits. Definitions of CAM providers vary between countries and organisations. In line with the Norwegian law about alternative treatment [9] we here define CAM providers as “providers others than authorized health personnel who give health-related treatment outside the established health services.” CAM provider visits may encompass any conceivable CAM treatment, however, the most commonly used CAM providers offer massage, acupuncture, naprapathy, reflexology and spiritual healing [10]. Chiropractors are authorised health personnel in Norway [11], and thus not included as CAM providers in this study. The use of psychiatric outpatient services in this study is defined as visits to private and public providers, including visits to hospital staff like nurses and social workers, supervised by psychiatrists and psychologists.

The utilisation of CAM providers has increased in Europe in recent years [12]. Utilisation in Norway is higher among women than men and higher in younger/middle ages [13, 14]. Patients visit CAM providers due to a desire to achieve a more holistic view, active participation, and empowerment in care [15, 16], trust in CAM providers [15], distrust in traditional health care [17], and negative communication experiences with doctors [18].

All Norwegian citizens are provided a regular GP. The municipalities run first line medical services. Specialist services, consisting of hospitals and outpatient clinics, are run by regional health enterprises mainly owned by the state. Access to specialist care is usually achieved by referral from the GP (the gate-keeper role), however, waiting lists for psychiatric specialist consultations have been long.

Norway has universal insurance, and GP and specialist outpatient visits are co-payed by a small fee. CAM provider visits are fully paid by the users, and referral from the GP is not required.

Tromsø is the largest city in North Norway with around 72,000 inhabitants, and around 50 CAM providers (unpublished observation by AEK). The municipality is almost equal to Norway for key parameters like employment and unemployment, average gross income per capita, proportion of disability pensioners, number of physicians per 10,000 residents, and proportion of the population living in urban areas, whereas the population is younger and higher educated than the Norwegian average [19]. Tromsø hosts the University Hospital of North Norway with somatic and psychiatric services.

Our first study aim was to explore the level to which people with self-reported anxiety and/or depression use CAM providers, and to what extent CAM providers and psychiatric outpatient services are used separately or in combination. Secondly, we aimed to investigate whether the use of CAM providers, and the use of CAM providers and psychiatric outpatient services in combination, was associated with gender, age, education, income and self-rated degree of anxiety/depression.

## Methods

### Study population

Questionnaire data were retrieved from the cross-sectional sixth Tromsø Study (Tromsø 6), consisting of two comprehensive self-administered questionnaires, clinical examinations and laboratory tests, conducted from October 2007 to December 2008. Four groups were invited; every resident aged 40–42 or 60–87 years (*n* = 12,578), a 10 % random sample of individuals aged 30–39 (*n* = 1056), a 40 % random sample of people aged 43–59 (*n* = 5787) and all subjects who had attended the second visit of the fourth Tromsø Study, if not already included in the other three groups (*n* = 341).

The first questionnaire was mailed with the invitation about 2 weeks ahead of the suggested appointment time. Participants were invited to attend whenever suitable within the survey opening hours (between 09:00 and 18:00). Non-respondents were given one reminder. The second questionnaire was handed out at attendance, and most participants completed it while waiting for the clinical examination. The comprehensive Tromsø 6 data include self-reported demographic and socio-economic characteristics, information about symptoms and diseases, health status, and use of medicines and health services. Since residents with severe mental disorders are unlikely to participate in population-based surveys like Tromsø 6 [20] the study mainly includes persons with minor psychiatric morbidity.

**Fig. 1:**
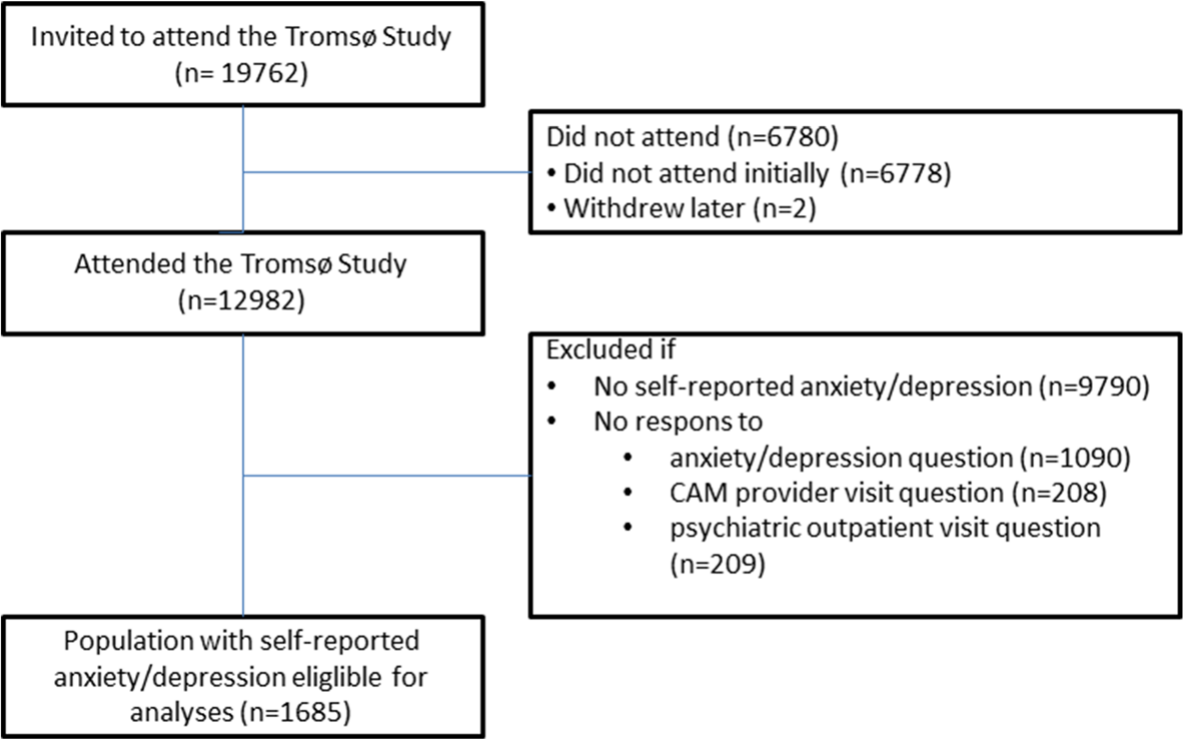
Flow chart of study population

Starting from the 12,982 participants, we first excluded those who reported no anxiety/depression (*n* = 9790), followed by participants who failed to inform about anxiety/depression (*n* = 1090), CAM visits (*n* = 208), and psychiatric outpatient visits (*n* = 209). The sample finally consisted of 1685 participants (Fig. 1).

### Measures

The three dependent dichotomous variables were use of CAM providers, use of psychiatric outpatient services, and use of both these services in combination once or more during the previous year (yes/no). Independent variables were age in 20-year groups, education, income, and self-reported degree of anxiety/depression. We defined three education response categories from the original five: low (primary and part of secondary school), middle (high school) and high education (college or university). The income variable referred to the household’s total gross income in the previous year. Eight original response categories were merged into low income (< NOK 200,000), low middle income (NOK 201,000–400,000), high middle income (NOK 401,000–700,000) and high income (> NOK 700,000). Anxiety/depression was categorised moderate or severe.

### Analyses

Data were analysed by means of descriptive statistics and logistic regressions. Correlations were tested with Spearman’s correlation coefficients. We constructed three multivariable regression models, one for each of the dependent variables. Identical sets of independent variables were introduced collectively into the models.

We used 95 % confidence intervals (CI) as significance level throughout the study. All analyses were accomplished using Stata, version 14.0.

## Results

In total 12,982 persons aged 30–87 years participated in Tromsø 6, constituting an overall response rate of 65.7 %. Eligible for analyses in our study were the 1685 persons who reported to suffer from anxiety and/or depression (Fig. 1). Women, persons aged 50–69 years, persons with high education, high middle income, and moderate anxiety/depression made up the largest groups (Table 1). A moderate degree of self-reported anxiety/depression was far more common than a severe anxiety/depression (Table 1).

**Table 1:** Sample characteristics (%)

	Both genders	Women (64.4 %)	Men (35.6 %)
Age	*n* = 1685	*n* = 1085	*n* = 600
30–49	33.6	31.6	37.2
50–69	48.6	48.3	49.1
70–87	17.8	20.1	13.7
Education^a^	*n* = 1659	*n* = 1072	*n* = 587
Low	33.6	35.9	29.5
Middle	32.0	32.5	31.2
High	34.4	31.6	39.3
Household income^b^	*n* = 1563	*n* = 982	*n* = 581
Low	17.7	21.5	11.2
Low middle	31.8	32.5	30.8
High middle	32.7	30.6	36.1
High	17.8	15.4	21.9
Degree of anxiety/depression	*n* = 1685	*n* = 1085	*n* = 600
Moderate	97.3	96.9	98.0
Severe	2.7	3.1	2.0

^a^Low (primary/part of secondary school), Middle (high school), High (college/university)

^b^Low (<200,000 NOK), Low middle (201,000–400,000 NOK), High middle (401,000–700,000 NOK), High (>700,000 NOK)

During the previous year, 17.8 % of people with anxiety/depression visited a CAM provider once or more, 11.8 % visited psychiatric outpatient services, and 2.5 % visited both (Table 2).

**Table 2:** Proportion of patients with self-reported anxiety/depression visiting CAM providers, psychiatric outpatient services, or both, once or more during the previous year

	CAM provider visits		Psychiatric outpatient visits		CAM provider and psychiatric outpatient visits	
	n/N	%	n/N	%	n/N	%
Total sample	300/1685	17.8	199/1685	11.8	42/1685	2.5
Gender						
Female	234/1085	21.6	140/1085	12.9	31/1085	2.9
Male	66/600	11.0	59/600	9.8	11/600	1.8
Age						
30–49	108/566	19.1	94/566	16.6	18/566	3.2
50–69	149/819	18.2	79/819	9.7	19/819	2.3
70–87	43/300	14.3	26/300	8.7	5/300	1.7
Education^a^						
Low	81/558	14.5	54/558	9.7	9/558	1.6
Middle	101/531	19.0	54/531	10.2	12/531	2.3
High	115/570	20.2	86/570	15.1	21/570	3.7
Household income^b^						
Low	42/276	15.2	40/276	14.5	10/276	3.6
Low middle	87/498	17.5	52/498	10.4	12/498	2.4
High middle	108/511	21.1	46/511	9.0	9/511	1.8
High	48/278	17.3	43/278	15.5	10/278	3.6
Degree of anxiety/depression						
Moderate	289/1639	17.6	178/1639	10.9	36/1639	2.2
Severe	11/46	23.9	21/46	45.7	6/46	13.0

^a^Low (primary/part of secondary school), Middle (high school), High (college/university)

^b^Low (<200,000 NOK), Low middle (201,000–400,000 NOK), High middle (401,000–700,000 NOK), High (>700,000 NOK)

Men with anxiety/depression visited CAM providers less likely than women (odds ratio [OR] 0.40, confidence interval [CI] 0.30–0.55), whereas higher educated people were more likely to visit than the lowest educated (OR 1.47, CI 1.02–2.13) (Table 3). The use of CAM providers was not associated with the degree of anxiety/depression (Table 3).

**Table 3:** Probability of visiting CAM providers, psychiatric outpatient services, or both once or more during the previous year in a population with self-reported anxiety/depression (multivariable logistic regressions)

	CAM provider visits (*n* = 1508)		Psychiatric outpatient visits (*n* = 1508)		CAM provider and psychiatric outpatient visits (*n* = 1508)	
	OR	95 % CI	OR	95 % CI	OR	95 % CI
Gender						
Female^a^	1.00	-	1.00	-	1.00	-
Male	**0.40**	**0.30–0.55**	0.72	0.51–1.02	0.63	0.31–1.29
Age						
30–49^a^	1.00	-	1.00	-	1.00	-
50–69	0.97	0.73–1.30	**0.59**	**0.42–0.84**	0.87	0.44–1.74
70–87	0.84	0.52–1.34	**0.34**	**0.18–0.63**	0.73	0.23–2.28
Education^b^						
Low^a^	1.00	-	1.00	-	1.00	-
Middle	1.28	0.89–1.83	1.04	0.66–1.66	1.32	0.51–3.42
High	**1.47**	**1.02–2.13**	**1.71**	**1.08–2.71**	2.47	0.98–6.25
Household Income^c^						
Low^a^	1.00	**-**	1.00	**-**	1.00	-
Low middle	1.16	0.76–1.77	**0.59**	**0.36–0.97**	0.65	0.26–1.63
High middle	1.42	0.91–2.22	**0.46**	**0.27–0.78**	0.45	0.16–1.27
High	1.08	0.64–1.83	0.71	0.40–1.26	0.78	0.26–2.33
Degree of anxiety/depression						
Moderate^a^	1.00	**-**	1.00	**-**	1.00	**-**
Severe	1.76	0.83–3.74	**7.09**	**3.51–14.34**	**7.53**	**2.75–20.65**

*OR* odds ratio, *CI* confidence interval

^a^Reference groups

^b^Low (primary/part of secondary school), Middle (high school), High (college/university)

^c^Low (<200,000 NOK), Low middle (201,000–400,000 NOK), High middle (401,000–700,000 NOK), High (>700,000 NOK)

Statistically significant findings are marked in bold

Regarding visits to psychiatric outpatient services there were no statistically significant gender differences, whereas the probability of visits were significantly reduced by higher age and household income (Table 3), and increased by higher education (OR 1.71, CI 1.08–2.71). The use of psychiatric outpatient services was strongly associated with a more severe anxiety/depression (OR 7.09, CI 3.51–14.34).

For those who used both CAM providers and psychiatric specialist services during the previous year, only a more severe degree of anxiety/depression was strongly associated with use (OR 7.53, CI 2.75–20.65) (Table 3).

There were no strong correlations (defined as rho > 0.5) between any of the independent variables in the models.

## Discussion

### Key findings

The main finding of the current study is that 17.8 % of people with anxiety/depression visited a CAM provider once or more during a year, 11.8 % visited psychiatric outpatient services, and 2.5 % visited both. CAM providers were more likely visited by women and by people with higher education. The probability of visiting psychiatric services, and CAM providers and psychiatric services in combination, was strongly associated with more severe symptoms of anxiety/depression, whereas the separate use of CAM providers was not.

### CAM provider visits

The CAM provider visit rate of 17.8 % in the current study is not far from a US study (data from 1997 to 98) where 20 % of those with anxiety attacks had visited a CAM provider within the last 12 months [7]. However, our CAM visit rate of 23.9 % among those with severe disease is somewhat higher than the 19.3 % visit rate among patients with severe depression [7]. Another US study (data from 1996) reported that only 9.8 % of those with a mental condition had visited a CAM practitioner [6], whereas an Australian study (data collected in 2007-08) found that 41.8 % of those with a chronic mental health condition had visited a CAM provider during the previous year [8]. The lower rates in the studies from the 90s conform with the general increased use of CAM providers during the period up to our survey [12]. In addition, a plausible explanation of the difference in visit rates is the inter study variation of definitions and methodology, for instance the inclusion or exclusion of chiropractors as CAM practitioners [6–8, 21]. Differences in availability and access to CAM providers, conventional psychiatric care, and other sources of mental care in different countries, geographical contexts, and health care systems might also influence the differences in CAM visit rates [22].

We found a higher use of CAM providers among people with anxiety/depression than among the general Tromsø population (12.7 %) [13], a pattern also observed in other populations [23]. Possible explanations might be easier access and less stigma when visiting CAM providers compared to conventional care [24, 25], the holistic perspective and active patient participation offered by CAM therapists [15, 16], and the higher somatic morbidity among people with mental health problems [26].

### Combined CAM provider and psychiatric specialist visits

Most CAM therapy use seem to be concurrent to the use of conventional treatment [7, 23, 27]. In the present study, only 2.5 % reported visits to both CAM providers and psychiatric services during the previous year. This is a notably low rate compared to the finding by Simon et al that CAM providers were aware of concurrent conventional care for mental health problems in 20–50 % of visits [21]. However, we studied the combination of CAM provider and conventional *specialist* care, whereas Simon et al also included conventional primary care. Despite these methodological differences, our low rate probably reflects a low access to psychiatric specialist care in Tromsø, Norway, as reported elsewhere [28]. In addition, the low rate might be related to stigma, leading people not to seek care in specialist psychiatric settings [29, 30]. Another possible explanation is that some people with anxiety/depression might be satisfied with care from one provider, whether care is offered by a CAM provider or a mental care specialist.

### Use according to gender and education

In the present study, men with anxiety/depression used CAM providers significantly less than women, which is in line with others’ findings [23]. Low use among men might be explained by preconceptions of masculine behaviour in a traditional sense, hindering men from showing their need for help and support [31]. Another explanation is related to the idea that men perceive their body and health as more “mechanical” than women, and that they, therefore, are less attracted to CAM where wholeness, communication and personal relations are more pronounced than biological mechanisms [32].

Overall, it is reported that CAM therapy users with depressive disorders have a higher level of education than non-users [23]. In line with this, we found that higher educated people more likely visited CAM providers. However, this contrasts findings of no such association in general populations in Norway [14, 33, 34], but conforms with most international studies [35]. It is believed that higher education increases the perception of mental problems and the willingness to seek care [28]. People with higher education might also be more able to find relevant information about CAM, and to afford such treatment [36].

There were no statistically significant associations between gender and education on the one hand, and the combined use of CAM providers and psychiatric specialist services on the other. This confirms with our previous study regarding use of psychiatric specialist services among people with anxiety/depression [28].

The current findings regarding age and household income are discussed elsewhere [14, 28].

### Use according to severity of disease

People with moderate anxiety/depression used CAM providers more than they used psychiatric specialists, whereas we found the opposite regarding people with severe disease. Still, less than half of those with severe anxiety/depression visited psychiatric specialist services during a year. Results regarding visits to psychiatric specialist services are discussed elsewhere [28].

In the group with severe anxiety/depression, 13 % visited both a CAM provider and psychiatric specialist services, and the probability of visiting was 7.53 times higher than among those with moderate disease. The severe sufferers thus seem to use CAM providers and conventional care additionally. The higher use of both services in patients reporting more severe depression is in line with Adams et al [37], but in contrast to Druss et al who found no difference regarding the degree of mental health problems [6].

Only 2.2 % of those reporting moderate anxiety/depression used combined care. This might be due to the overall lower use of both CAM and conventional care in the current study. Other reasons could be that people with moderate disease would avoid seeking help from conventional psychiatric services due to fear of stigma and feelings of guilt and shame [38], and also that CAM providers might be a substitute or an alternative pathway when access to conventional care is limited [39, 40]. On the other hand, those who report moderate ailments might be satisfied with CAM provider treatment alone. The line between prevention and treatment might be intertwined in many of these cases. A low threshold CAM service could be a proper supplement for some with minor morbidity, seeking to prevent worsening of symptoms.

Summing up, one might say that psychiatric specialist services seems to be reserved for those with the most severe disease, in keeping with the guidelines that specialist care should treat the sickest, and that moderate ailments to a greater extent are treated elsewhere. However, our findings add to a solid documentation that the use of mental health services both in general populations and in people with anxiety/depression in high income countries is limited, indicating that these symptoms are undertreated [2, 41, 42].

### Strengths and limitations

Particular strengths of this study were the large sample size, the high response rate, and the comprehensive coverage of information about health, disease, and socio-economic status in the questionnaires.

Nevertheless, the study should be interpreted in light of some limitations. Despite a high baseline response rate, our sample may not be entirely representative of the population suffering from anxiety/depression, as it is well known that women, healthier persons, and higher socio-economic groups are more likely to participate in population surveys [43]. In Tromsø 6, attendees were older, and the proportion women were higher than in non-attendees [44, 45]. In the second Tromsø Study (1979-80) the participation of people with psychiatric morbidity was approximately 20 % lower than for those without such morbidity [20], and lower participation is likely the case for Tromsø 6 as well. However, this applies particularly to serious psychiatric morbidity [20, 46].

Additionally, our data might underestimate psychiatric morbidity and treatment seeking due to perceived stigma [29], and treatment seeking might also be underestimated in the population since questions about psychiatric conditions and use of services were spread throughout the questionnaire, probably increasing inaccuracies [47]. However, there is hardly any reason why people should report anxiety/depression but not use of CAM providers and psychiatric services, thus the relative validity between these variables should be quite robust.

The validity of self-reported data as such may be questioned, although agreement between self-reported and registered health care utilisation is generally high [48]. It might also be easier to report anxiety/depression in a self-administered questionnaire than reporting to health care providers. Moreover, self-reported anxiety/depression might be the best available measure for our study purpose, since research based on doctor made diagnoses would make it difficult to include the non-visitors.

Our analyses focused on anxiety/depression, but we cannot rule out the possibility that participants may have had other psychiatric and/or somatic ailments or diseases in addition, because the reasons for visiting were not reported.

Furthermore, it might be a problem that we asked about anxiety/depression at the time of the survey, whereas health care utilisation was reported for the previous 12 months. However, the onset of these diseases is often ahead of 30 years of age [3, 4], making it unlikely that this have affected our study.

Finally, we cannot exclude the possibility of unmeasured confounders of the reported associations.

## Conclusions

During a year, around 17.8 % of people with anxiety/depression visited a CAM provider whereas only 2.5 % visited CAM providers and psychiatric services in combination. People with severe disease seem to use CAM providers and psychiatric services additionally, whereas those with moderate disease seem to use these services as alternative pathways. Our results indicate that the most severe sufferers use psychiatric specialist services more than those with moderate disease, and that CAM provider treatment might be a substitute for conventional care, particularly in patients with moderate disease. We suggest treatment outcome and efficacy in the different contexts to be an important direction for future research.

## Abbreviations

CAM: Complementary and alternative medicine
CI: Confidence interval
GP: General practitioner
NOK: Norwegian Kroner
OR: Odds ratio
Tromsø 6: The sixth Tromsø study
